# Apoptin enhances the oncolytic properties of vaccinia virus and modifies mechanisms of tumor regression

**DOI:** 10.18632/oncotarget.2579

**Published:** 2014-11-19

**Authors:** Galina Kochneva, Evgeniy Zonov, Antonina Grazhdantseva, Anastasiya Yunusova, Galina Sibolobova, Evgeniy Popov, Oleg Taranov, Sergei Netesov, Peter Chumakov, Elena Ryabchikova

**Affiliations:** ^1^ Novosibirsk State University, Novosibirsk, Russia; ^2^ State Research Center of Virology and Biotechnology “Vector”, Koltsovo, Russia; ^3^ Institute of Chemical Biology and Fundamental Medicine, SB RAS, Novosibirsk, Russia; ^4^ Engelhardt Institute of Molecular Biology, Moscow

**Keywords:** Vaccinia virus, apoptin expressing recombinant, oncolytic properties, carcinoma A431

## Abstract

A recombinant vaccinia virus VVdGF-ApoS24/2 expressing apoptin selectively kills human cancer cells *in vitro* [Kochneva et al., 2013]. We compared the oncolytic activity of this recombinant with that of the parental strain L-IVP using a model of human A431 carcinoma xenografts in nude mice. Single intratumoral injections (2×10^7^ PFU/mouse) of the viruses produced a dramatic decrease in tumor volumes, which was higher after injection of apoptin-producing virus. The tumor dried out after the injection of recombinant while injection of L-IVP strain resulted in formation of cavities filled with cell debris and liquid. Both viruses rapidly spread in xenografts and replicate exclusively in tumor cells causing their destruction within 8 days. Both viruses induced insignificant level of apoptosis in tumors. Unlike the previously described nuclear localization of apoptin in cancer cells the apoptin produced by recombinant virus was localized to the cytoplasm. The apoptin did not induce a typical apoptosis, but it rather influenced pathway of cell death and thereby caused tumor shrinkage. The replacement of destroyed cells by filamentous material is the main feature of tumor regression caused by the VVdGF-ApoS24/2 virus. The study points the presence of complicated mechanisms of apoptin effects at the background of vaccinia virus replication.

## INTRODUCTION

Oncolytic viruses are characterized by the ability to recognize and selectively kill cancer cells. The ability to replicate preferentially in malignant cells is a property of many viruses belonging to a variety of viral families. Many experimental and clinical studies demonstrate a promising therapeutic potential of oncolytic viruses [2, 3, 4, 5] hence encouraging researchers to develop safer and more efficient oncolytic virus strains. The advantages of oncolytic viruses as anticancer therapeutics and the problems that are associated with this approach have been recently reviewed in details [6, 7, 8, 9]. The recombinant “armed” oncolytic viruses are being designed to carry additional therapeutic proteins that enhance the efficiency of specific destruction of the malignant cells. Such viruses not only destroy tumor cells during the replication, but also serve as delivery vehicles providing the release of therapeutic molecules inside the tumor. Successful applications of recombinant adeno-, herpes and orthopoxviruses have been recently summarized [10]. The effector molecules introduced into the viral genome aim either at the direct killing of cancer cells [11], or at an enhancement of antitumor immune responses [12]. Apoptin, a non-structural protein of the chicken anemia virus, is considered as one of the most promising effectors specifically inducing apoptosis of cancer and transformed cells [2, 13, 14]. The ability of apoptin to induce apoptosis has been demonstrated in more than 70 human cancer cell lines [2, 15]. As apoptin could be a promising tool that enhances the specific destruction of cancer cells, several apoptin-producing oncolytic viruses have been constructed and tested. Insertion of the apoptin gene into the genome of Newcastle disease virus [16] and Fowlpox virus [17] was shown to improve the oncolytic potentials. Apoptin-producing recombinants based on the adenovirus vector were capable of inducing apoptosis in cancer cells originating from bladder [18] and stomach [19]. Apoptin was successfully applied to kill SW480, HeLa and MCF-7 cancer cell lines using a lentivirus-based construct [21].

Vaccinia virus (VACV) is considered as one of the most promising oncolytic viruses for cancer therapy. It possesses a unique set of advantages, including the natural tropism for tumor cells, the ability to infect a wide spectrum of cells, rapid and exclusively cytoplasmic replication, and the large genome suitable for the insertion of multiple foreign genes [22, 23]. We constructed an apoptin-producing recombinant VACV based on the L-IVP strain [1]. In the construct the apoptin gene replaces a part of the *C11R* gene that encodes the viral growth factor (VGF). The deletion of the VGF gene provides an additional attenuation of the virus [24]. The obtained VACV recombinant VVdGF-ApoS24/2 effectively expresses apoptin in the infected cells, and demonstrates a significantly enhanced selective lysis of the human cancer cell lines A549, A431, U87MG, RD and MCF-7 *in vitro,* as compared with the parental virus strain L-IVP and its variant VVdGF2/6 with the deletion of the *C11R* gene [1]. The present study aims the assessment of oncolytic properties of the apoptin-producing recombinant VACV (VVdGF-ApoS24/2) in the model of nude mice xenografts of the human A431 epithelioid carcinoma cells, in comparison with the parental L-IVP virus strain.

## RESULTS

### Virus replication in A431 cells *in vitro* and *in vivo*

**Figure 1:**
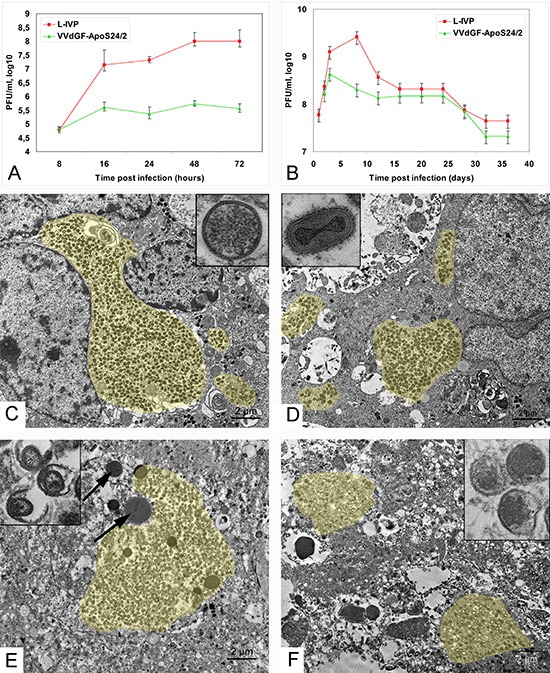
Vaccinia virus replication in carcinoma A431 cells **(A)** Titers of the L-IVP and VVdGF-ApoS24/2 strains in the A431 cells during 72 h of *in vitro* incubation, and **(B)** in tissue of the carcinoma A431 xenografts in mice. Data are represented as a mean ± SD. **(C)** Active replication of the L-IVP strain and **(D)** apoptin-producing recombinant VVdGF-ApoS24/2 strain in cells of the carcinoma A431 xenografts, 2 days after virus injection. Boxes show C) immature and D) mature viral particles at large magnification. **(E)** Destruction of infected cells in xenografts on day 8 after injection of the L-IVP strain, and **(F)** VVdGF-ApoS24/2 strain. Arrows are pointed at lipid droplets. Boxes show damaged viral particles in cell “remains” in tumors on day 55 after the injection with E) L-IVP strain, and F) VVdGF-ApoS24/2 strain. Viral accumulations are painted with yellow color. Transmission electron microscopy, ultrathin sections.

Both the L-IVP and the apoptin-expressing recombinant VACV strain VVdGF-ApoS24/2 have demonstrated the ability to replicate in the A431 carcinoma cells *in vitro*, although the yield of the recombinant virus was evidently lower (Fig. 1A). Similar pattern was revealed in the A431 carcinoma xenografts within 12 days after a single injection of identical doses of the L-IVP and the VVdGF-ApoS24/2 virus strains (Fig. 1B). The yield of the L-IVP virus in infected tumors exceeded the yield of the recombinant virus, as well as the maximum value of the virus titer. Examination of ultrathin tumor sections in the electron microscope two days after the injection of the L-IVP or VVdGF-ApoS24/2 viruses revealed an active replication of both virus strains (Fig. 1C, D). Groups of infected tumor cells were found mostly in the central regions of the tumor; the periphery was free of infected cells. The infected cells became swollen and showed typical signs of poxvirus replication: granular viroplasm, spherical immature and ovoid mature virions composed of membrane envelope and distinct nucleoid. Formation of the enveloped virus was observed in cells infected with any of the virus strains.

The viruses rapidly spread in the tumors, and by day 4 after the injection only few non-infected cells could be found at the tumor periphery, while the whole tumor consisted of infected cells. The observation correlates well with the increase of virus titers in the tumors (Fig. 1B). It should be noted that both VACV strains replicated exclusively in tumor cells: no signs of virus infection were detected in the endothelium and connective tissue inside the tumor, as well as in the tumor capsule and the adjacent connective tissue. Electron microscopy has shown that both L-IVP and VVdGF-ApoS24/2 viruses completely invaded the tumor and destroyed tumor cells within 8 days after the injection. The tumors were densely packed with cell debris and viral particles (Fig. 1E, F) and virtually resembled sacks filled with detritus, lipid droplets, immature and mature viral particles, and clumps of viroplasm. Having examined numerous ultrathin sections from different areas of the tumors we could not find a single cell maintaining its integrity. The virus titers within the tumor started to decrease from day 8 after the injection (Fig. 1B) indicating that the replication of both viruses stopped because all tumor cells have been destroyed. In accordance with the virus titration data, the examination of ultrathin tumor sections on days 2, 4 and 8 after injection indicates that the L-IVP strain produces more mature virions than the VVdGF-ApoS24/2 strain.

Although the destruction of tumor cells prevented the virus from further multiplication, infectious virions continued to be present in the tumor tissue for as long as 36 days after the injection (Fig. 1B). The experiment totally lasted for 55 days, however the titration of virus was impossible after day 40 because the tumor tissue became shrank and dry in mice that received the VVdGF-ApoS24/2 strain. The electron microscopy revealed a decrease of virus deposits in the tumor tissue during the experiment, and some damage of viral structure was evident at the late stages of the observation. No signs of viral replication in normal stroma cells within and around the tumors were detected during the whole period of observation (55 days), evidencing the high selectivity of the VACV replication in tumor cells.

### Apoptin production by the VVdGF-ApoS24/2 strain

**Figure 2:**
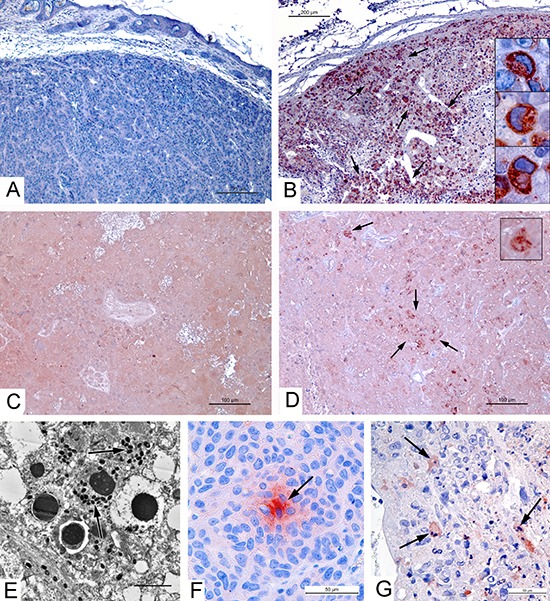
Apoptin production by VVdGF-ApoS24/2 strain and apoptosis in carcinoma A431 xenografts Immunohistochemical staining of paraffin sections for apoptin-connected FLAG peptide in tumor peripheral zones on day 4 after injection of: **(A)** saline, negative reaction; **(B)** VVdGF-ApoS24/2 strain, positive reaction; and in tumor central zones on day 36 after injection of **(C)** L-IVP strain, negative reaction; **(D)** VVdGF-ApoS24/2 strain, positive reaction. Arrows show the sites of positive reaction, boxes show positively stained cells at high magnification. **(E)** Apoptosis of infected cell in the xenograft ultrathin section, day 2 after injection with VVdGF-ApoS24/2 strain. Arrows show virus particles. **(F)** Positive reaction for Apaf-1 protein (is shown by arrow) in the xenograft cells, day 4 after saline injection. **(G)** Caspase-3 positive cells (are shown by arrows) in xenograft, day 4 after injection with VVdGF-ApoS24/2 strain. Paraffin sections after processing with specific antibodies were treated with AEC chromogen, counterstained with hematoxylin.

The expression of apoptin by the VVdGF-ApoS24/2 strain in the CV-1 cells *in vitro* was described in our previous report [1]. In order to confirm the production of apoptin *in vivo* we performed an immunohistochemical analysis of the A431carcinoma xenografts injected with the VVdGF-ApoS24/2 virus. To detect the expression of apoptin in paraffin sections of tumors we used antibodies specific to the FLAG peptide. Strongly positive immunostaining was detected in the sections of tumors injected with the VVdGF-ApoS24/2 virus (Fig. 2B) while the sections of tumors from the control mice and from the mice that received the L-IVP virus were stained negatively (Fig. 2A, C). The specific staining was attributed to a granular material in the cytoplasm while the nuclei remained unstained (Fig. 2B, boxes). We conclude that the apoptin expressed from the recombinant vaccinia virus has a cytoplasmic localization. Examination of sections from the tumor 36 days after injection of the VVdGF-ApoS24/2 virus revealed apoptin deposits despite the tumor destruction and the loss of cell structure integrity (Fig. 2D). By day 55 the tumor sections were negative for the apoptin-FLAG immunostaining (data not shown). Thus, replication of the VVdGF-ApoS24/2 virus in A431 cells *in vivo* coincided with the active production of apoptin, which stayed preserved inside the tumors for at least 36 days despite the destruction of tumor cells.

Obviously, we expected to find an increase of apoptosis in the tumors, which were injected with the VVdGF-ApoS24/2 strain expressing apoptin, in comparison with parental L-IVP strain. However, we failed to detect any noticeable difference in the number of apoptotic cells in ultrathin sections of infected zones in tumors injected with two viruses. Signs of apoptosis were observed in some infected cells (Fig. 2E), as well as in non-infected tumor cells. We applied immunostaining to evaluate amount of apoptotic cells in paraffin sections of the A431 xenografts injected with saline, VVdGF-ApoS24/2 and L-IVP strains. Dependence of apoptin induced apoptosis on Apaf-1 was shown previously [25], so we used this marker of apoptosis. The Apaf-1 protein (Fig. 2F), indicating formation of apoptosome in the process of mitochondrial pathway of apoptosis, was virtually absent in the sections of all tumors. Number of Apaf-positive cell foci was small: 1.1–1.5 per mm^2^ of xenograft section on day 2, and 0.6–1.0 on day 4 post injection with both viruses and saline. Probably, this result is related to mutant form of p53 gene in the A431 cells [1]. Immunostaining of another apoptosis marker, caspase-3 (Fig. 2G), was much more abundant and brighter in tumors injected with both VACV viruses, than in the sections of saline injected tumors. This result was rather expected than surprising, because caspase-3 is the effector enzyme operating in different pathways of apoptosis. The number of caspase-3 positive cells was 16±2.1, 77±3.7 and 74±4.0 per mm^2^ of tumor periphery 2 days after injection of saline, L-IVP and, and VVdGF-ApoS24/2 strains, correspondingly; and 10±2.4, 67±3.5 и 72±3.8 cells after 4 days. Thus, injection of both viruses evidently enhances the apoptosis in A431 xenografts, however number of apoptotic cells do not differ in tumors injected with apoptin-producing recombinant and parental VACV L-IVP strains. Taken together, our immunostaining study evidences for inability of apoptin, produced by recombinant VVdGF-ApoS24/2 virus, to induce apoptosis in A431 xenograft cells.

### The apoptin-expressing VACV affects the regression of tumor xenografts

**Figure 3:**
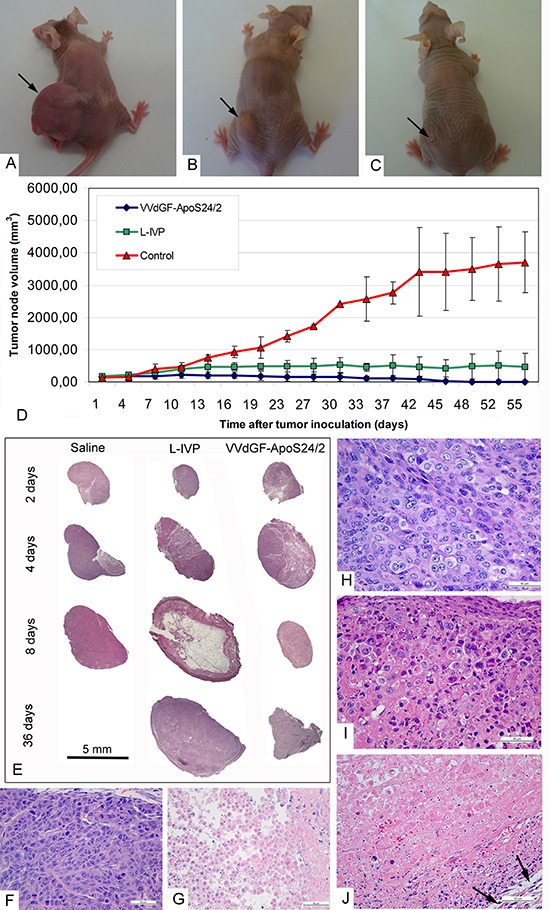
Regression and histological characteristics of A431 tumors in mice after single intratumoral injection of the VACV L-IVP or VVdGF-ApoS24/2 strains Photographs of mice on day 45 after injection of: **(A)** saline (control), **(B)** L-IVP strain, and **(C)** VVdGF-ApoS24/2 strain. **(D)** Tumor growth kinetics after injection of the L-IVP and VVdGF-ApoS24/2 strains in comparison with saline-treated mice. Data are represented as a mean ± SD. (E) Representative paraffin sections of the A431 tumors at different times after single injection of saline, the L-IVP and VVdGF-ApoS24/2 strains. Note large cystic cavity in the tumor on day 8 after the L-IVP strain injection. (F) Peripheral zone and (G) central necrotic zone of the A431 tumors in mice, 2 days after the injection of saline (control group). Peripheral zones of the tumors in mice after: **(H)** 2 days; **(I)** 4 and **(J)** 8 days after the injection of VVdGF-ApoS24/2 strain. Swollen rounded cells were few on day 2 and increased in number on day 4 when some necrotic cells were also observed at the tumor periphery. The tumor tissue was completely destroyed on day 8 after injection, hematoxyline stained nuclei debris is seen in adjacent to capsule, which is shown by arrows. Paraffin sections, hematoxyline and eosin staining.

Single injections of the parental L-IVP or the apoptin-expressing VVdGF-ApoS24/2 viruses in the A431 xenografts resulted in a notable reduction of tumor volumes as compared with the untreated tumors (Fig. 3A-D). The rates of the tumor volume regression in mice injected with the VVdGF-ApoS24/2 virus clearly outpaced those in mice received the parental L-IVP strain, and the difference was especially noticeable on day 35 after the injection (Fig. 3D). The tumors in mice treated with the control L-IVP virus were tense and springy at palpation. Visual observation often showed a liquid on the cuts of the L-IVP virus injected tumors. In contrast, the tumors were substantially shrank or completely disappeared in mice treated with the VVdGF-ApoS24/2 virus. At the late stages of experiments the tumors were hardly detectable by palpation, resembling a small sealing at the tumor area, dry and hardly visible on the cuts.

The differences between the tumors injected with the two VACV strains were clearly noticeable by light microscopy of histological sections (Fig. 3E). Substantial cystic cavities were present in the tumors 8 days after the injection with the L-IVP virus while the VVdGF-ApoS24/2 virus-injected tumors were packed with a dense unstructured material and kept the appearance up to the end of the experiment. A detailed histological examination at high magnification has revealed that the control untreated tumors (Fig. 3F, G) were enclosed in a capsule composed of fibroblasts and collagen fibers. Tumor lobules were separated by thin interlayers of loose connective tissue containing blood vessels. Each tumor had a necrotic zone, which occupied 10–25% of the tumor area. Histological structure of the tumors was severely affected by the injection of the VACV strains. The area of necrosis increased up to 50% on day 4 after the virus injection, and on day 8 the tumor structure was completely destroyed and the whole tumor volume was filled with the detritus. Although an alteration of blood vessel cells was observed, the vessels kept their integrity. The process of tumor destruction proceeded along with the virus spreading, and usually the peripheral zone of the tumor was affected at later stages. Figures 3H-J demonstrate the sequential steps of the tumor tissue destruction by the VVdGF-ApoS24/2 virus.

A single injection of the parental L-IVP or the apoptin-expressing VVdGF-ApoS24/2 viruses into xenografts of the A431 cells was sufficient for the complete destruction of tumor cells within 8 days. Notably, the process of tumor destruction was not associated with an inflammatory reaction in the tumor tissue. We observed the signs of congestion in blood vessels of the affected areas of tumors on day 2, and slight accumulation of neutrophils in the lumen of blood vessels on day 4 after the virus injection. The neutrophils and other leukocytes were not found outside the blood vessels within 4 days. A slight increase in the number of neutrophils was noted in the blood vessels as well as in the capsule and connective tissue surrounding the tumor on day 8 after the virus injection, although inflammatory infiltrates were not found. Few macrophages were dispersed within the destroyed tumor tissue, they contained multiple large phagosomes filled with cell debris. No leukocytes were detected in the tumors or in the adjacent tissue at the late stages of the experiment. The absence of the inflammatory reaction could be explained by an immune deficiency of the athymic mice used in this work.

**Figure 4:**
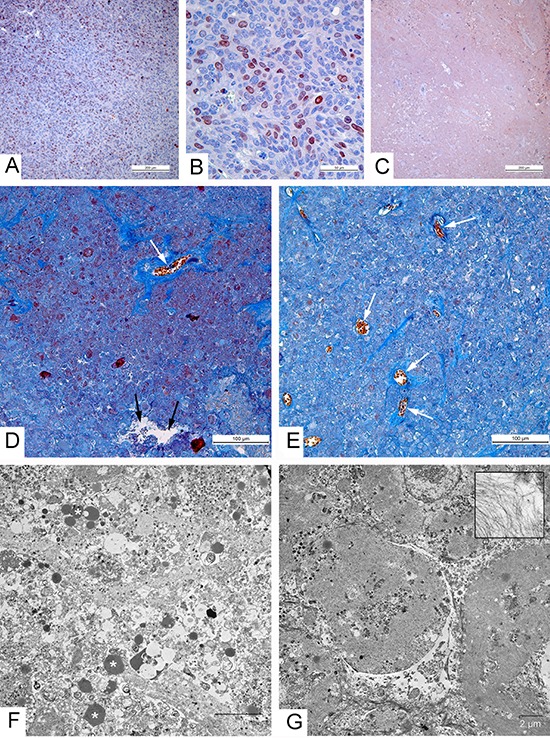
Morphological characteristics of carcinoma A431 xenografts in mice **(A-C)** Immunohistochemical reaction for the Ki-67 protein. (A) High proliferative activity of tumor cells in xenograft injected with saline; (B) Positive reaction for the Ki-67 protein in nuclei of carcinoma A431 cells, 2 days after injection with the L-IVP strain; (C) Negative staining in tumor, indicating absence of proliferating cells on day 36 after injection with VVdGF-ApoS24/2 strain; paraffin sections, AEC chromogen, counterstaining with hematoxylin. **(D-G)** 36 days after injection of the viruses. (D, E) Paraffin sections, Picro-Mallory staining. (D) Cell debris in the tumor injected with the L-IVP strain, (E) unstructured material in tumor injected with VVdGF-ApoS24/2 strain. Both photos: blue staining indicates collagen fibers, black arrows show cavity and white arrows show blood vessels; brown clumps represent cell debris stained by hematoxiline. (F, G) Representative electron micrographs showing the same areas of tumors injected with L-IVP strain (F) and VVdGF-ApoS24/2 strain (G). Masses of cell debris are present on photo F, lipid droplets are shown by asterisks. Photo G shows cell “shades” separated with streaks of cell debris. The box shows filaments at large magnification.

Visual observations and tumor measurements clearly showed different patterns of the tumor regression after the injection of the two virus strains and we examined tumor samples collected on days 36 and 55 after the treatment. Having taken into account the fact that all tumor cells were destroyed within 8 days after the virus injection, we checked the presence of live tumor cells using an immunohistochemical detection with antibodies to Ki-67. This protein is a marker of dividing cells (Fig. 4A, B) that is commonly applied for the detection of proliferating cells and for the cancer prognosis [25, 26]. No specific staining was found in paraffin sections of the tumors 36 and 55 days after the injection with the control L-IVP and with the recombinant viruses (Fig. 4C). The destruction of tumor tissue at the late stages of the experiment was evident (Fig. 4D-G).

Examination of the paraffin sections on days 36 and 55 after the injection of the two VACV strains revealed clear differences between the tumors. In the case of the L-IVP virus the tumor volumes were filled with cell debris and contained empty cavities and collagen fibers that apparently originated from the tumor stroma and blood vessels, the remains of nuclei appeared as brown clumps (Fig. 4D). Electron microscopy of the same tumors revealed cell debris and numerous lipid droplets scattered on electron lucent background (Fig. 4F). The picture was consistent with the flooding of the tissue and the liquid observed on tumor cuts. Meantime, examination of paraffin sections of tumors injected with the VVdGF-ApoS24/2 virus revealed a relatively dense unstructured material containing distinct bundles of collagen fibers and contours of blood vessels (Fig. 4E). The amount of cell debris dramatically reduced compared to that observed in the tumors injected with the parental L-IVP virus. Electron microscopy revealed the deposits of filaments that formed structures resembling the shades of cells that held their shape. Small areas of cell debris were observed between the deposits of filaments (Fig. 4G). The filaments were 7–10 nm thick and supposedly represented the actin and intermediate filaments. Similar filaments were scattered between the cell debris in the tumors injected with the L-IVP virus, however the accumulations of filaments were not observed. Thus, we found characteristic changes in the cytoskeleton of tumor cells after the injection of the VVdGF-ApoS24/2 virus and these changes accompanied the shriveling of the tumor tissue.

### Cytoskeleton alterations in the VACV infected A431 carcinoma cells

**Figure 5:**
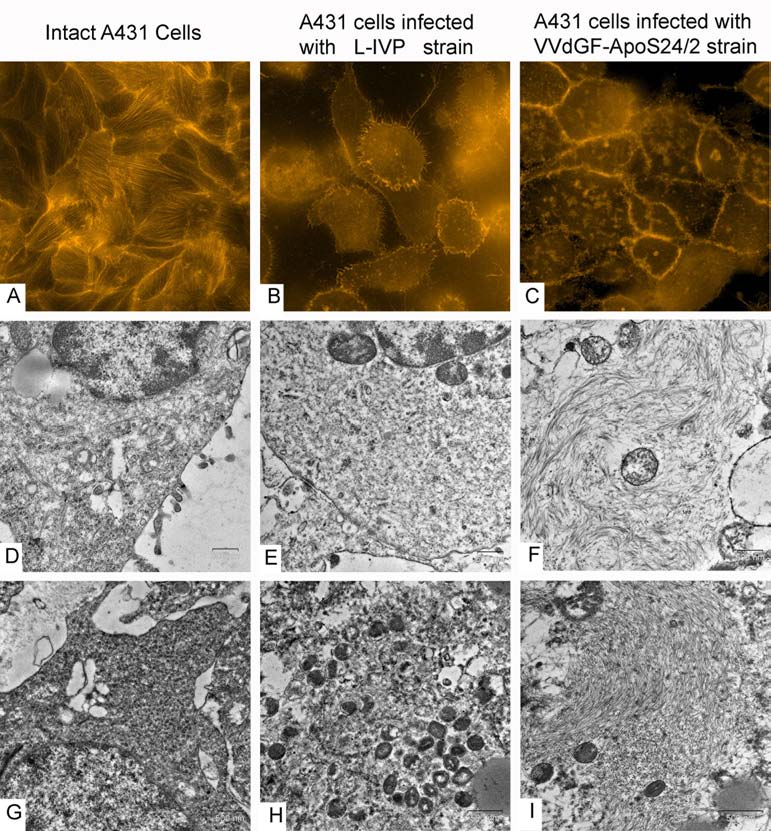
Disorganization of cytoskeleton in carcinoma A431 cells caused by VACV strains *in vitro* and *in vivo* The upper row shows actin filaments visualized in fluorescent microscope with TRITC-falloidin in: **(A)** intact cell culture, **(B)** L-IVP strain infected cells, and **(C)** VVdGF-ApoS24/2 strain infected cells. The middle row shows the same cells visualized in electron microscope: **(D)** intact cell, **(E)** infected with the L-IVP strain, and **(F)** VVdGF-ApoS24/2 strain, clumps of actin are seen. Photos A-F were obtained from A431 cells *in vitro*, 48 h after the infection with VACV. The bottom row shows electron microscopic images of the A431 cells *in vivo*: **(G)** a cell of saline-treated tumor, **(H)** a tumor cell containing debris and viral particles, 36 days after injection of the L-IVP strain, **(I)** a tumor cell containing an accumulation of filaments, 36 days after the injection of the VVdGF-ApoS24/2 strain.

To visualize changes in the cytoskeleton following the *in vitro* infection of A431 cells with the VVdGF-ApoS24/2 and L-IVP viruses, we stained actin with the TRITC-falloidin for the fluorescent microscopy. We found that the viruses alter the cellular architectonics differently. Small clumps of actin were observed at the periphery of the L-IVP virus infected cells while in the cells infected with the VVdGF-ApoS24/2 virus the actin filaments formed distinct clumps of larger sizes in the cytoplasm (Fig. 5A-C). By electron microscopy we also found the accumulation of filaments in the cells infected with the VVdGF-ApoS24/2 virus (Fig. 5D-F). Similar changes were observed on ultrathin sections of the A431 tumor xenografts *in vivo*, the cells demonstrated a distinct accumulation of filaments after injection of the VVdGF-ApoS24/2 virus (Fig. 5G-I).

## DISCUSSION

In the present study we have tested the effect of apoptin on the oncolytic activity of the vaccinia virus. We compared the regression of carcinoma A431 xenografts after a single injection with the identical doses of the apoptin-expressing recombinant VVdGF-ApoS24/2 and parental the L-IVP strain, and found that the recombinant provides a more efficient destruction of the tumor. It was reasonable to expect that the enhancement of the oncolytic activity was determined by the apoptotic death of tumor cells induced by apoptin expressed by recombinant VVdGF-ApoS24/2 strain, similar to previously reported other apoptin-producing recombinant viruses [16, 18, 19]. However, examination of the apoptosis by electron microscopy and immunostaining of the Apaf-1 and caspase-3 clearly demonstrated that both recombinant and parental strains have induced an identical insignificant level of apoptosis in the A431 carcinoma xenografts. We assumed that the action of apoptin derived from the expression of the recombinant VVdGF-ApoS24/2 strain could be somehow altered. The cytoplasmic localization of apoptin detected by the immunostaining in the tumor xenografts was a sign of the alteration.

Previous studies showed that in cancer cells apoptin localizes to the nuclei, but it has a cytoplasmic localization when expressed in normal cells [28]. It was proposed that the nuclear localization of apoptin in cancer cells is important for their preferential killing [29]. However, the data about the subcellular localization of apoptin in the cells of tumors injected with the apoptin-producing recombinant viruses are scarce. The nuclear localization of apoptin has been demonstrated in the gastric cancer cells SGC-7901 infected *in vitro* with the apoptin-expressing recombinant adenovirus AdHu5 [19]. The presence of apoptin in the xenografts of A549 cells was detected following the injection of the apoptin-producing recombinant of Newcastle disease virus, but the subcellular location of apoptin was not tested [16]. Regarding the apoptin-producing poxviruses there is only one report on a Fowlpox virus recombinant providing the evidences of apoptosis in cancer cells [17], but no data on the apoptin subcellular localization have been presented. Vaccinia virus is a lytic virus that rapidly and irreversibly alters all cellular synthesis [30]. Apoptin expressed by the VVdGF-ApoS24/2 virus should operate on the background of the processes of viral synthesis and maturation. We suppose that the alteration of cellular metabolism by the virus replication does not allow apoptin to realize its apoptotic killing of the tumor cells and the cytoplasmic localization represents an element of this inability.

In order to understand the mechanisms of enhanced oncolytic effect of the VVdGF-ApoS24/2 strain, we have examined its replication in comparison with the parental L-IVP strain. We have found that after the injections to xenografts of the A431 carcinoma cells both viruses actively replicate in tumor cells although the virus yield, as well as the maximum value of the virus titer inside the injected tumors, was higher in the parental L-IVP strain compared to the recombinant expressing apoptin. The difference in viral production could be explained by the disruption of the *C11R* gene and by the insertion of the apoptin gene in the VVdGF-ApoS24/2 strain leading to a decreased replication [24]. Thus, the VVdGF-ApoS24/2 strain replicates less efficiently in the A431 cells, and so the enhancement of oncolytic properties may not be related to the virus replication. The oncolytic properties of the VACV have been revealed in many studies [31, 32, 33, 34], however no data on the infectivity of the virus produced inside the tumors have been reported. Besides, it is unknown for how long a virus can stay live inside the injected tumors. In our experiments both the L-IVP and the apoptin-producing VVdGF-ApoS24/2 viruses have demonstrated the ability to replicate actively in tumor cells and to maintain the infectious potential for at least 36 days after the virus injection. The viruses infected tumor cells with high specificity and stayed inside the tumors for as long as 55 days.

Undoubtedly, the complete destruction of tumor cells by replicating the VVdGF-ApoS24/2 and the L-IVP strains is the main effector mechanism of viral oncolysis. These viruses showed the ability of fast tumor destruction, and no differences were found between the tumors injected with the viruses using the applied methods within 8 days. The subsequent events of tumor regression unfolded in the already destroyed tumors clearly differed in mice received recombinant VVdGF-ApoS24/2 or parental L-IVP viruses: drying of the tumor in the first case and flooding in the second. In fact, these two pathways represent modifications of cell death induced by the viruses, because no signs of the “host response” have been found in the xenografts. The main feature of tumor regression caused by the VVdGF-ApoS24/2 virus was the emergence of the filamentous material, which replaced destroyed tumor tissue, made the tumor dry, and indicated the cytoskeleton involvement in the process of tumor regression.

The alteration of cytoskeleton by a poxvirus infection was reported earlier [35, 36]. Undoubtedly, the formation of actin deposits in the A431 cells infected with the VVdGF-ApoS24/2 virus is attributed to apoptin. The interaction of apoptin with the elements of cytoskeleton β- actin, α- and β-tubulin in H1299 cells was reported earlier [37], but the mechanisms of interaction were not identified. Probably apoptin or apoptin-bound molecules block the depolymerization of actin and thereby induce the formation of actin accumulations. These accumulations fill the main part of tumor volume while the fraction of debris is substantially reduced. A question arises: what happens to the cell debris in tumors injected with the VVdGF-ApoS24/2 strain?

We suppose that apoptin directs the tumor destruction to the way of dehydratation and shrinkage. The apoptin being unable to induce “complete” apoptosis, possibly could trigger some early apoptosis events in tumor cells. The cytoplasmic accumulation of apoptin observed in the virus-infected tumor cells could affect some cytoplasmic pathways of cell death thereby securing the complete lysis of cell organelles, elimination of debris, and dry shriveling of the tumor. Replication of poxviruses in the cells represents a very complicated process which is not completely understood yet, and the death of infected cells is the least studied. Thus, the ovarian carcinoma cell death after the infection with the Lister strain of the VACV appeared as a complicated interplay of the programmed events related to necrosis, apoptosis and autophagy, and the authors concluded that in these cells the Lister strain induces a programmed necrosis [38]. In the other study the Western Reserve strain of the VACV was capable of inducing apoptosis in the malignant HeLa cells, but not in the immortalized BSC-40 cells while the Dryvax and Praha strains of the VACV were found to induce apoptosis in both HeLa and BSC-40 cells [39]. Therefore, the existence of the virus-strain specific induction of cell death should be taken into consideration when testing the effects of new oncolytic virus strains.

The results of the present study demonstrate that a single injection of the VVdGF-ApoS24/2 virus provides a more efficient destruction of the A431 carcinoma xenografts than the parental L-IVP strain although the effect is not related to the death of tumor cells via apoptosis. This finding suggests the existence of diverse mechanisms of antitumor action of the apoptin-producing recombinant viruses. It is interesting to know whether the other apoptin-producing recombinant viruses could produce the shriveling of different tumors as it was caused by the VVdGF-ApoS24/2 strain injected to the A431 carcinoma xenografts. Our study highlights the need for a careful examination of any new recombinant virus in order to reveal particular mechanisms and pathways by which the virus mediates its anti-tumor effect.

In summary, an enhancement of the virus-mediated oncolysis by the construction of apoptin-producing recombinants has been reported for the Newcastle disease virus [16], the adeno-associated virus [20], adenoviruses [40] and the fowlpox virus [17]. We have examined the mechanisms of oncolytic effect of the apoptin-expressing recombinant VACV strain VVdGF-ApoS24/2 in comparison with the parental L-IVP virus strain. A single injection of either of the viruses into the A431 carcinoma xenografts resulted in a substantial decrease of the tumor volume evidencing for a high oncolytic activity of the viruses. However, the apoptin-expressing recombinant virus produced a more efficient regression of the tumors, despite a lower production of the infectious virus. Apoptin produced by the recombinant VACV localizes to the cytoplasm, in contrast to the previously described preferentially nuclear localization of apoptin in cancer cells. The discrepancy could be explained by an alteration of certain cellular pathways by the VACV infection. The expression of apoptin correlated with an aggregation of actin filaments in the A431 cells both *in vitro* and in mouse xenografts while in the L-IVP infected cells actin was found in the depolymerized state. The main features associated with the tumor regression following the injection of VVdGF-ApoS24/2 recombinant were the shrinkage of the tumors and the disappearance of cell debris inside the tumors observed at the microscopic level. We conclude that apoptin expressed by the recombinant VACV does not induce a typical apoptosis, but rather modifies the virus-induced cell death in such a way that the tumors shrink without the excessive formation of cell debris and exudate. Undoubtedly, the shrinkage of the tumor is more desirable effect of the treatment than the flooding and accumulation of cell debris. The study provides evidence of the enhancement of oncolytic properties of vaccinia virus using the insertion of the apoptin gene and points the presence of the complicated mechanisms of apoptin effects at the background of the VACV replication.

## METHODS

### Cell cultures and viruses

Human epidermoid carcinoma cell line A431 and monkey kidney fibroblast cell line CV-1 were obtained from the American Type Culture Collection (ATCC; Manassas, VA). Cells were cultured at 37°C with 5% CO_2_ in Dulbecco's modified Eagle's medium (DMEM, Invitrogen, USA) supplemented with 10% of fetal bovine serum (FBS, HyClone, USA) and antibiotics (100 U/ml penicillin/100 U/ml streptomycin).

Vaccinia virus, Lister strain (L-IVP, Institute for Virus Preparations, Moscow, Russia) was obtained from the State Collection of Viral and Rickettsial Disease Agents of the State Research Center of Virology and Biotechnology “Vector”.

The replication-competent recombinant VACV producing apoptin (VVdGF-ApoS24/2 strain) was obtained by insertion of the synthetic chicken anemia virus gene encoding apoptin, a selective killer of cancer cells, into the VACV (strain L-IVP) genome. The insertion replaces a major part of the viral *C11R* gene encoding the VGF. The construction of the VVdGF-ApoS24/2 strain and its ability to express apoptin and to kill a variety of cancer cells *in vitro* were described earlier [1]. The viruses were propagated in the CV-1 cells, purified by a sucrose gradient (25–45%) sedimentation, sonicated and titrated using the plaque assay in the CV-1 cell monolayers. Virus titers were expressed as plaque forming units (PFU) per ml. The viral stocks represented 10^9^ PFU/ml in sterile saline and aliquots were stored at −80°C.

To study the virus growth characteristics, the monolayer of A431 cells was infected with 0.01 PFU of the L-IVP or with recombinant viruses incubated at 37°C for 48 h, with three sequential freezing and thawing cycles. The obtained virus suspension was sonicated and titrated by the plaque assay.

### Sampling of infected cells for microscopic studies

For electron microscopy the monolayers of A431 cells were infected with 0.01 PFU per cell of the L-IVP or recombinant virus, incubated at 37°C for 24 and 48 h, and sampled by scrapping and centrifugation (3000 g, 5 min). Pellets were fixed with 4% paraformaldehyde at 4°C for 24 h.

For fluorescent microscopy the A431 cell monolayer was grown on glass coverslips (Cover glass, BDH) and infected with 0.01 PFU per cell of the L-IVP or recombinant virus incubated at 37°C for 24 and 48 h. At the indicated intervals post infection cells were washed with the PBS and fixed with 4% paraformaldehyde in the PBS at room temperature (RT) for 20 min. Cells fixed on coverslips were washed three times with the PBS and permeabilized with 0.1% Triton X-100 in the PBS. To detect polymerized actin the specimens were treated with tetramethylrhodamine B isothiocyanate (TRITC)–phalloidin (Sigma, P 1951) diluted at 1:1500 for 30 min, RT. Phalloidin-stained coverslips were washed three times with the PBS, embedded in 50% glycerol solution in PBS, and stored at 4°C. Fluorescent microscopy was performed using AxioImager Z1 supplied by cooling CCD-camera AxioCam MR, and filters 02, 10, 15 (ZEISS). Digital images were acquired using the program package AxioVision 4.

### Animal model and virotherapy

Female *Nu/Nu* mice (Nursery for Laboratory Animals, Institute of Bioorganic Chemistry, Moscow, Russia) were used for the xenograft model of the human A431 carcinoma. The mouse studies were performed under protocols approved by the SRC VB VECTOR Institutional Animal Care and Use Committee (NIH Office 85 of Laboratory Animal Welfare, Number A5505-01).

Tumors were generated in 8-10 week *Nu/Nu* mice (20–26 g) by subcutaneous injections of 5 × 10^6^ A431 cells (in 100 μl PBS) in the left hind region. Tumor volume was measured in two dimensions using a digital caliper and calculated with the following formula: [(length × width^2^ × 0.5] [17]. When tumors reached 175–211 mm^3^ in volume (10 days after tumor cells injection), the mice received a single injection of the VACV L-IVP or VVdGF-ApoS24/2 (2 × 10^7^ PFU/mouse in 100 μl of saline (0.9% NaCl) into the tumor. Mice of the control group received 100 μl of saline. The tumor growth was monitored every 3–4 days after the injection. Mice were sacrificed at different intervals (2–55 days). Tumors and surrounding tissues were dissected, sampled for the virus titration, and fixed in 4% paraformaldehyde for light and electron microscopy.

Virus titers in tumors were tested as follows: tumor samples in triplicates for each mouse group were homogenized in Hanks' solution to prepare 10% suspension (v/v) at the indicated intervals after the infection. Samples were freeze-thawed three times, sonicated for 30 s and cleared by centrifugation at 2000 g for 20 min. Virus titers were determined from cleared supernatants by standard plaque assay in the CV-1 cells.

### Light microscopy and immunohistochemistry

The samples for light microscopy were routinely processed using Sakura Tissue-Tek II (Sakura, Japan) machine and embedded in paraffin. Sagittal sections (3–4 μm) of the whole tumors were prepared and routinely stained with hematoxylin and eosin, or Picro Mallory Trichrome (Bio-Optica, Italia), then they were embedded in Bio Mount medium (Bio-Optica, Italia). Paraffin sections for immunohistochemical analysis were mounted on the polylysine-coated slides (Thermo Scientific, USA). Processing with the antibodies to Ki-67, caspase-3 and Apaf-1 was carried out according to the manufacturer's protocols (Abcam, Great Britain). Ki-67 is a nuclear protein serving as a marker of proliferating cells, which appears in late G1-phase, S-, G2- and M-phases of the cell cycle, and is absent in the G0-phase [41, 42]. Apaf-1 (apoptotic peptidase activation factor 1) is a cytosolic marker of apoptosis mitochondrial pathway [43]. To evaluate apoptin production in cancer cells its C-terminal region was tagged with the FLAG epitope [1]. Commercial mouse monoclonal antibodies for the FLAG Octa-Probe H-5,(Santa-Cruz, CA) were used to detect apoptin by immunohistochemistry in paraffin sections of the A431 tumors. The product of all immunohistochemical reactions was visualized using the AEC Single Solution detection system (Abcam, Great Britain), final staining of the sections was performed with Erlich's hematoxylin.

Paraffin sections were examined with the light microscope Leica DM 2500 supplied with the digital camera Leica DFC420C (Leica, Germany).

### Electron microscopy

Tumors fixed in 4% paraformaldehyde were sliced into 1–2 mm thick sections and postfixed in 1% osmium tetroxide solution, routinely processed and embedded into a mixture of epon-araldite (SPI, USA). Semithin sections were prepared from hard blocks, stained with Azur 2 and examined in the light microscope for the selection of areas for the ultrathin sectioning. At least four blocks of both central and peripheral parts of each tumor on each time-point were used to study the ultrastructure. Ultrathin and semithin sections were cut using Leica EM UC7 ultramicrotome (Leica, Germany), routinely contrasted by uranylacetate and lead citrate (SPI, USA), and examined with the transmission electron microscope JEM 1400 (Jeol, Japan). The images were collected by the side-mounted digital camera Veleta (SIS, Germany).

### Statistical analysis

Mann-Witney U test was used to determine significance of the results. A *P* value of <0.05 was considered as significant. Statistical significance was determined at P < 0.05.
